# Diagnosis of pericardial cysts using diffusion weighted magnetic resonance imaging: A case series

**DOI:** 10.1186/1752-1947-5-479

**Published:** 2011-09-24

**Authors:** Asam Raja, Jonathon R Walker, Maneesh Sud, Joe Du, Matthew Zeglinski, Andrew Czarnecki, Negareh Mousavi, Davinder S Jassal, Iain DC Kirkpatrick

**Affiliations:** 1 Department of Radiology, University of Manitoba, Winnipeg, Manitoba, Canada; 2 Institute of Cardiovascular Sciences, St Boniface General Hospital, University of Manitoba, Winnipeg, Manitoba, Canada; 3 Section of Internal Medicine, Department of Internal Medicine, University of Manitoba, Winnipeg, Manitoba, Canada; 4 Section of Cardiology, Department of Internal Medicine, Bergen Cardiac Care Centre, St Boniface General Hospital, University of Manitoba, Winnipeg, Manitoba, Canada

## Abstract

**Introduction:**

Congenital pericardial cysts are benign lesions that arise from the pericardium during embryonic development. The diagnosis is based on typical imaging features, but atypical locations and signal magnetic resonance imaging sequences make it difficult to exclude other lesions. Diffusion-weighted magnetic resonance imaging is a novel method that can be used to differentiate tissues based on their restriction to proton diffusion. Its use in differentiating pericardial cysts from other pericardial lesions has not yet been described.

**Case presentation:**

We present three cases (a 51-year-old Caucasian woman, a 66-year-old Caucasian woman and a 77-year-old Caucasian woman) with pericardial cysts evaluated with diffusion-weighted imaging using cardiac magnetic resonance imaging. Each lesion demonstrated a high apparent diffusion coefficient similar to that of free water.

**Conclusion:**

This case series is the first attempt to investigate the utility of diffusion-weighted magnetic resonance imaging in the assessment of pericardial cysts. Diffusion-weighted imaging may be a useful noninvasive diagnostic tool for pericardial cysts when conventional imaging findings are inconclusive.

## Introduction

Congenital pericardial cysts arise when a portion of the pericardium pinches off during embryonic development [1,2]. The majority of pericardial cysts are found in the right anterior cardiophrenic angle. They often lack internal septations and fail to enhance with contrast [3]. Pericardial cysts typically contain a simple fluid whose attenuation on computed tomography (CT) is similar to water. Their contents are usually hyperintense on T2-weighted magnetic resonance images (MRI) images and hypointense on T1-weighted signals [3].

The diagnosis of pericardial cysts is not always straightforward since they may present in atypical locations [3]. Moreover, their elevated protein content may increase their density on CT images, decrease their T2-weighted MRI signals and increase their T1-weighted signals [3]. As a result, differentiating these lesions from hematomas or neoplasms can be quite challenging. The lack of internal architecture may differentiate a cystic lesion when findings on CT and conventional MRI sequences are equivocal. However, this method is not always reliable [3].

Diffusion-weighted imaging (DWI) using MRI is able to differentiate the diffusion restriction of protons within a tissue by calculating the apparent diffusion coefficient (ADC) [4]. The diffusion of protons within a simple cyst is less restricted when compared to a variety of more complex and particularly malignant lesions [4]. Simple cysts, as a result, display larger ADC values [4] which can be utilized as a diagnostic tool in order to differentiate a pericardial cyst from other pericardial lesions.

## Case Series

### Case 1

**Figure 1:**
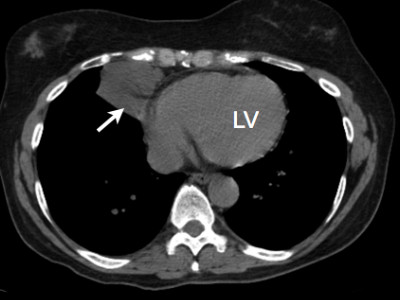
**Case 1 -Axial CT image of this patient's thorax demonstrates a lesion in the right anterior cardiophrenic angle**. The attenuation of the contents measured 19.6 Hounsfield Units (HU), or near water density.

**Figure 2:**
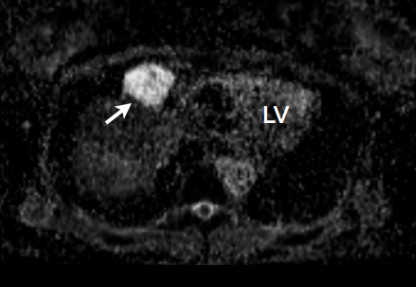
**Case 1 -The ADC map using DWI CMR demonstrates a high value of the cyst contents, 3.47 × 10^-3 ^mm^2^/s**. The ADC of cerebrospinal fluid measured in this patient was 3.1 × 10^-3 ^mm^2^/s.

A 51-year-old Caucasian woman was referred for assessment of chest pain and dyspnea. Her past history was significant for cervical dysplasia. A physical examination was unremarkable. A twelve-lead electrocardiogram showed normal sinus rhythm. A subsequent exercise treadmill test did not reveal any evidence of stress-induced ischemia. Her left ventricular systolic function was normal as demonstrated by transthoracic echocardiography (TTE). Multidetector CT (MDCT) identified a fluid density lesion measuring 6 × 4 cm at the right anterior cardiophrenic angle, consistent with a pericardial cyst (Figure 1). Our patient underwent cardiac magnetic resonance imaging (CMR) for further assessment of this lesion. DWI performed at b-values of 0s/mm^2^, 50s/mm^2^, 400s/mm^2 ^and 800s/mm^2 ^demonstrated a steep drop in signal from the cyst contents with increasing b-values corresponding to an ADC value of 3.47 × 10^-3^mm^2^/s (Figure 2).

### Case 2

A 66-year-old Caucasian woman with past history of hypertension and diabetes mellitus presented with a long-standing history of chest pain and shortness of breath. Physical examination and a twelve-lead electrocardiogram were unremarkable. Multiple cardiac imaging studies including a TTE and myocardial perfusion study did not show any evidence of ischemia. MDCT revealed a cyst within her anterior mediastinum measuring 4.7 × 1.7 cm, representing a possible pericardial cyst in an atypical location. CMR was performed to further evaluate this lesion. DWI demonstrated signal characteristics consistent with free diffusion within the cyst and an ADC of 3.02 × 10^-3^mm^2^/s.

### Case 3

A 77-year-old Caucasian woman with a past medical history of vitamin B12 deficiency and cholecystectomy underwent MRI for evaluation of suspected biliary colic. An incidental finding of a 10.4 × 4.2 cm cystic lesion along the right cardiac border was suspected to be of pericardial origin. Our patient was referred for further characterization of the lesion with CMR. The calculated ADC within the cyst was 3.18 × 10^-3^mm^2^/s.

## Discussion

Congenital pericardial cysts are rare, yet important, lesions that account for 7% of all mediastinal masses [1]. The prevalence of pericardial cysts is one in 100,000 [1] and approximately 60% of patients present between 30 and 50 years of age [2]. Pericardial cysts are commonly located in the left (51% to 70%) and right (28% to 38%) cardiophrenic angles. A small percentage, however, are located in the upper mediastinum, hilus or cardiac border (8% to 11%) [5]. The classic description of a pericardial cyst is a 1 cm to 5 cm unilocular, smooth-walled cyst with an outer layer of endothelial or mesothelial cells [6]. Their serous fluid-filled center and lack of solidity distinguishes them from other pericardial masses. Rare complications such as infection and hemorrhage may, however, confound efforts to characterize pericardial cysts using this description [6].

Up to one third of patients with pericardial cysts will become symptomatic at some point [1,7]. Atypical chest pain, persistent cough or new onset dyspnea secondary to the cyst's mass effect on adjacent structures are frequent presenting symptoms of patients with pericardial cysts [1,7]. In rare, yet devastating occasions, pericardial cysts may spontaneously rupture or hemorrhage into surrounding tissues leading to cardiac tamponade, heart failure and sudden death [8-10]. Thus, an early and accurate diagnosis in symptomatic individuals is necessary in order to offer prompt and potentially life-saving therapy.

Pericardial cysts are usually discovered incidentally as an unexpected round mass on routine chest radiography or TTE in asymptomatic patients [1,6]. On TTE, a pericardial cyst appears as a homogeneous echolucent mass, which is consistent with minor attenuation of the ultrasound through a low-density fluid-filled structure. There also exists an echo-free space indicating its separation from the cardiac chambers [6]. The differential diagnosis is broad and includes tumors undergoing cystic degeneration, such as Hodgkin disease, germ cell tumors, mediastinal carcinomas, nerve root tumors, abscesses and pancreatic pseudocysts [1,6]. The current standard of care mandates follow-up CT with intravenous contrast or CMR (T1- and T2-weighted methods) to confirm the diagnosis of a pericardial cyst.

Cardiac CT has proven uses for characterizing pericardial masses. Its accuracy, however, suffers from similar pitfalls as chest radiography and echocardiography. It cannot distinguish malignant tissue from non-malignant fluid-filled cysts with a great degree of confidence [6]. Similarly, T1- and T2-weighted MRI may also provide inconclusive results when cysts contain proteinaceous, non-serous fluid [6]. Thus, there is a lack of a reliable, non-invasive imaging modality that can differentiate pericardial cysts from other pericardial masses with similar appearances, but substantially different prognoses and treatments.

Differentiating exudate from transudate on MRI has previously been reported using DWI and ADC values. Under optimized parameters, DWI is an effective tool with a high sensitivity and specificity (91% and 85% respectively) for discriminating fluids with different protein and cellular contents [11]. Moreover, DWI seems to be a reliable tool for differentiating other benign chest-mediastinal masses [12], focal breast lesions [13] and bladder lesions [14] from malignant lesions. Application of DWI's discriminatory power to fluid-filled, pericardial lesions is a logical next step.

The present case series illustrates three independent patients in whom pericardial cysts displayed consistently high ADC values. ADCs may thus prove useful in differentiating symptomatic pericardial cysts from neoplastic and infectious mediastinal lesions that are otherwise irreconcilable by conventional CT or MRI. Future studies, with surgical confirmation, are warranted to evaluate the utility of diffusion weighted MRI as the first test of choice for the noninvasive assessment of pericardial cysts.

## Conclusion

This report presents three cases of pericardial cysts that were evaluated with DWI using CMR. The ADC maps consistently demonstrated high ADC values, indicating free diffusion of protons within the pericardial cysts. This study is a first attempt to investigate the utility of DWI in the assessment of pericardial cysts. Further study into the diagnostic utility of DWI when CT and MRI are equivocal in patients with a pericardial mass is warranted.

## Consent

Written informed consent was obtained from the patients for publication of this case series and its accompanying images. A copy of the written consent is available for review by the Editor-in-Chief of this journal.

## Competing interests

The authors declare that they have no competing interests.

## Authors' contributions

All authors contributed to the writing of the manuscript. All authors read and approved the final manuscript.
